# Dr. Wood, on Vaccine Inoculation

**Published:** 1806-02

**Authors:** J. Wood

**Affiliations:** Newcastle upon Tyne


### *To the Editors of the Medical and Phyſical Journal.*

Gentlemen,

It is now twelve months since I sent you a Report of the Progress and Success of Vaccination in this town, particularly at the Dispensary; and I now proceed to give you another report, with that satisfaction which the mind experiences in the investigation and gradual attainment of truth.

In my former report I endeavoured to shew, that the vaccine disease was a security against the small-pox at the distance of three and four years from the period of inoculation of the former, to that of a second inoculation with the latter.

That the vaccine disease was a security against the small-pox, although the pustule had been prevented by too much inflammation from assuming the usual regular appearances.

That the eruption peculiar to infants did not prevent vaccination being a security against the small-pox, although present when vaccination took place.

And lastly, That after the *system* had been *vaccinated*, a second inoculation with the vaccine matter will not produce a pustule similar in its stages, or its appearance, to that produced by the first inoculation; and that a second inoculation with vaccine matter was a preferable test of security to inoculation with small-pox matter. Time has amply confirmed these several positions, not only in my own experience and observations, but in that of others; perhaps I may except my second and third position as confined to my own observations, the first and fourth have been very ably confirmed and enlarged upon by Dr. Pearson's great experience and abilities.

In addition to what I have stated in my second and third positions, as to the security afforded by vaccination against small-pox, not being lessened by too much inflammation destroying the order of the pustule, or by the presence of *strophulous* during the vaccination, I can also now, with some confidence, advance my opinion, though contrary to some late prevailing opinions, that some other eruptions, *herpes* for instance, do not prevent, nor lessen this security. I have been cautious in advancing this opinion, though founded on my experience and frequent observation, because Dr. Jenner has entertained a different opinion. I have, on the contrary, been accused by a Mr. Clement, in No. 75 of your Journal, of not producing in my former report, those " sterling instructions as well as observations," on this particular point, that Dr. Jenner had given. I apprehend that those instructions are only *sterling*, which are the result of much and accurate observation, or that those observations only are of value that are derived from experience. Of my observations, therefore, my experience warrants me in concluding that the presence of *herpes* neither prevents nor lessens the security usually obtained by vaccination against the small-pox; so far from my former report being deficient in " sterling instruction, I conceive it was the reverse, by not holding out an opinion contrary to my observation.

Among the patients vaccinated at the Dispensary here, we have now an ample field of observation, and I would not on this point have depended entirely even on my own observations, so far as to have advanced the opinion I have done, if the experience of Mr. Anderson, Surgeon to the Dispensary, and of Mr. Wilkie, the resident Surgeon and Apothecary, had not as much as possible tended to confirm it; amongst others, Mr. Wilkie took me to a child that had been vaccinated, which had herpetic eruptions in large spots in several parts of its body, and in particular at the sides of its mouth, and on one arm very near the pustule of vaccination; and notwithstanding the progress and appearance of the pustule, were no way affected, and the usual security was obtained. It is remarkable that all herpetic eruptions inflame with the progress of inflammation in the vaccine pustule, and scab with it also; in my last report, I mentioned a case given to me by Mr. Fife, surgeon, in which which the herpetic eruption and the vaccine pustule kept pace exactly in the period of inflammation, in the time of both continuing to discharge prior to scabbing, and in that of scabbing and healing. This child, on a second inoculation, was found to have been before sufficiently vaccinated. Mr. Anderson, Surgeon to the Dispensary, in answer to my inquiry of him on this subject, says, "I have inoculated some children with vaccine matter at the period when the skin was *covered* with an herpetic eruption, but I do not recollect that there was any difference in the formation of the vaccine pustule on the arm; it appeared to proceed much in the same state as if there had been no eruption on the skin." That these children were securely vaccinated will appear in the sequel of this report. On this part of my subject I have only to add, that it has been long remarked that vaccination shewed itself in the system very evidently by affecting, for a short time, the surface or discharge of any sore, or wound, on any part of the body; and having observed, that *herpes* scabbed and healed with the vaccine pustule, I was about to propose vaccination for the cure of a very obstinate case of herpes on the face and breast of a young woman, a patient of mine, when great increased action took place in the septan, in consequence of an abscess in one of the extremities, and removed the herpetic affection entirely.

The progress of vaccination in this town and neighbourhood has been beyond expectation during the last twelve months, "vires acquirit eundo," and the success of it equal to the most sanguine expectation. In the three first years, 1801, 2, 3, nine hundred and twenty-one were vaccinated at the Dispensary; in 1804, six hundred and thirty-seven; and in 1805, to December 2, one thousand seven hundred and eight, being more than the four former years altogether.

| | |
|---|---|
| In 1801, 2, 3 - - | 921 |
| 1804 - - - - | 637 |
| 1805 - - - - | 1708 |
| Total | 3266 to Dec. 2, 1805. |

I feel not a little elevated in being able to say, that not one of these 3266 have taken the small-pox, although it has been raging in every part of this town and neighbourhood for fifteen months past, the vaccinated children having stood amidst the general wreck untouched and uninjured. In a village near this town, Swalwell, I am informed formed by Mr. Anderson, surgeon, that about thirty children have died of small-pox, and a lady resident in it has taken pains to make the most accurate inquiries, and has found that every vaccinated child in the village has escaped, though surrounded (it being but a small village) with the contagion of small-pox. Thus, by the judicious and unremitting exertions of Mr. Anderson and Mr. Wilkie, of the Dispensary, have many valuable lives been saved during this great epidemic; they could only have had the stimulus of the public good at heart, for the office has been far from pleasant; in it they have had much to encounter and to surmount; even the labour itself has not been inconsiderable, when they have sometimes inoculated more than 100 in one day; the success of inoculation here has therefore been gratifying in the extreme. The public confidence in it, as a security against the small-pox, was somewhat shaken by the cases that had been published in the south, and the reports that had been circulated even here; and I began to think of contradicting them in a public manner in the early part of this year; for though hitherto I had always, in all my concerns, never thought it necessary to contradict reports in a formal manner, on the maxim *that truth must ultimately prevail*, yet I now think error should be attacked as it rises, not only that it may not, like the organized system of France, overpower the cause of truth, but also as life is too short to wait for the slow prevalence of truth when resting only on its own buoyant powers. To return from this digression, I was about to contradict the reports unfavourable to cow-pox, when I found the *popular opinion* of it increasing daily in its favour, by the increasing numbers brought for inoculation to the Dispensary, which I considered more forcible in its favour than any thing I could say; even in winter last, and also this summer, they have not ceased, as formerly, to bring their children for inoculation during the very cold and very warm weather, which circumstance did not only shew the increased confidence of the public in the cow-pox, but also that they were glad to fly from the danger of the small-pox, and were convinced that warm weather did not make the children more affected during vaccination. So many children coming from the very contagion of small-pox to be vaccinated, has given opportunities of noticing many extraordinary circumstances as to the time when vaccination gives the resistance to the small-pox contagion, and also as to the striking security it affords; there have been repeated instances of two children in the same family inoculated lated with cow-pox on the same day, and the one sickening with small-pox a few days after, before the security was afforded by vaccination, and the other going through the cow-pox and constantly remaining with the other under small-pox, even in the same bed, without being in the least affected; while the one in whom small-pox appeared before the system was affected with cow-pox, had its sufferings diminished as soon as the mild influence of the cow-pox prevailed. But as my observations so exactly agree with those of Dr. Pearson, lately given to the public,* I therefore think it superfluous to repeat them here; indeed, so definitive and so able are the reports of Dr. Pearson, that I will henceforth consider them as superceding the necessity of any further report from me, unless I find that either any of my observations do not agree with those of Dr. Pearson, or that any of mine have not been noticed by him. Dr. Jenner's well-merited fame I can neither detract from, nor add to; but I must be allowed to say, that we are greatly in debt to Dr. Pearson (and let me not omit Mr. Ring) for establishing the laws of the cow-pox; and without whose exertions I almost fear that in this country it would have suffered, *at least*, a temporary suspension of its fame.

On reviewing the success of vaccination in this town and neighbouring villages, it appears to have been greater than that reported to you of cow-pox inoculation in Shields, by Dr. Winterbottom. I have stated to you the success of it in the Dispensary,† and I have not heard of any one instance

* Extract from the Minutes of the Original Vaccine-Pock Institution, Medical and Chirurgical Review for November last, (Article Miscellaneous) p. lxxv.

† In the Medical and Chirurgical Review for March last, (Article Miscellaneous, p. cvi.) it is mentioned that a boy named Stevens, residing at No. 23, Millbank Street, Westminster, had had the small-pox after being vaccinated at the Dispensary here; *no such name* appears on the books of the Dispensary here as secure against the small-pox; only those names were then inserted that had gone through the disease in a satisfactory manner, by being seen by the surgeon after inoculation; the names of all those children that were inoculated and did not return for approbation on inspection were omitted; whether Stevens was of that number cannot now be ascertained, and therefore as a check in future, the name of every child inoculated at the Dispensary *will be recorded;* and the number not ascertained to be secure, or, in other words, not known to have gone regularly through the disease, will be given under a separate head.*

* *The Editors are much obliged to Mr. Wood for suggesting this regulation, which they hope will be adopted in all public institutions.*

instance of failure in private practice; and I dare say, the number inoculated by private practitioners in this town must have been *at least* equal to the number inoculated at the Dispensary. I was informed by one gentleman, a surgeon in this town, that he had had a case of small-pox after cow-pox at the interval of three weeks only; but it is evident, if the proper constitutional affection had taken place, the security would have lasted three weeks; the only doubt I ever heard, was that of Mr. Goldson's, of the security not lasting for three years or upwards; besides, no case of this kind can have any weight in the public opinion, unless it had been seen by many witnesses.

It appears very desirable to ascertain whether there is any cause for the greater success of inoculation for the cow-pox as a security against the small-pox in this town, than in many other places; I have never been able to guess at any other cause than one, viz. the invariable practice here of inoculating in *both* arms. I certainly observe, in many reports, that inoculation has only taken place in *one* arm; most assuredly, the constitutional affection affords the only security against small-pox, and without doubt the chance of the constitutional affection is greater from *two* inoculations than from *one;* in my own mind, I have always considered some test of the constitutional affection, as the first object in vaccine inoculation; and where such is not certain, I consider the inoculation in *both* arms as next to a certainty, until the attainment of this object. I dare say many have been deceived in supposing the constitutional affection to have taken place, when it has not, for in this way only can I suppose the vaccine inoculation ever to have failed as a security against small-pox. I lately knew an instance of a child taking small-pox shortly after inoculation with small-pox matter; because the inoculation was *local* only, no eruption took place from it.

In order to ascertain the success of vaccination here as a security against small-pox, we have not merely been satisfied with *not hearing* of any miscarriages, but the most active enquiries have always been made to find out any instance of failure; we have both negative and positive evidence, therefore, on this important point.

That *one* death has not taken place from vaccine inoculation in the Dispensary is not a matter of wonder, but that *not one* death should have *occurred* during the period of inoculation in the number of 3266 children is rather a matter of surprise to me, as there are certainly many chances of children children dying during that period from dentition and other diseases, in a much less given number. I have not heard of any death but one shortly *subsequent* to cow-pox inoculation, and that proceeded from a distinct disease three weeks after inoculation. I have also observed with much pleasure, that scrofula does not arise after cow-pox, as it certainly did after small-pox. I have never yet seen on the extensive charity of the Dispensary, and on the equally extensive one of the Infirmary here, any case of scrofula after cow-pox; in my experience, it is less frequent amongst children than formerly.

I will now bring this long report to a conclusion. Perhaps the papers in your Journal, *under the head of vaccination,* do not draw that attention now they did at first; but they remain half contented and half ignorant, who *give up* a matter of the first consequence to mankind in the *middle* of the subject; and they only think right, who do not allow their zeal to abate while any part of it continues open for interesting inquiry.

I am, &c.

*Newcastle upon Tyne,*

*Dec.* 12, 1805.

J. WOOD, M.D.

